# Biotic and abiotic drivers of tree seedling recruitment across an alpine treeline ecotone

**DOI:** 10.1038/s41598-018-28808-w

**Published:** 2018-07-18

**Authors:** Esther R. Frei, Eva Bianchi, Giulietta Bernareggi, Peter Bebi, Melissa A. Dawes, Carissa D. Brown, Andrew J. Trant, Steven D. Mamet, Christian Rixen

**Affiliations:** 1 WSL Institute for Snow and Avalanche Research SLF, Flüelastrasse 11, 7260 Davos Dorf, Switzerland; 2 Swiss Federal Institute for Forest, Snow and Landscape Research WSL, Zürcherstrasse 111, 8903 Birmensdorf, Switzerland; 3 Institute of Terrestrial Ecosystems, Department of Environmental Systems Science, ETH Zurich, Universitätstrasse 22, 8092 Zurich, Switzerland; 4 Dipartimento di Bioscienze, Università di Parma, Parco Area delle Scienze 11/A, 43124 Parma, Italy; 5 Department of Geography, Memorial University, 230 Elizabeth Avenue, St John’s, NL A1B 3X9 Canada; 6 School of Environment, Resources and Sustainability, University of Waterloo, 200 University Avenue West, Waterloo, ON N2L 3G1 Canada; 7 Department of Soil Science, University of Saskatchewan, 51 Campus Drive, Saskatoon, SK S7N 5A8 Canada

## Abstract

Treeline responses to climate change ultimately depend on successful seedling recruitment, which requires dispersal of viable seeds and establishment of individual propagules in novel environments. In this study, we evaluated the effects of several abiotic and biotic drivers of early tree seedling recruitment across an alpine treeline ecotone. In two consecutive years, we sowed seeds of low- and high-elevation provenances of *Larix decidua* (European larch) and *Picea abies* (Norway spruce) below, at, and above the current treeline into intact vegetation and into open microsites with artificially removed surface vegetation, as well as into plots protected from seed predators and herbivores. Seedling emergence and early establishment in treatment and in control plots were monitored over two years. Tree seedling emergence occurred at and several hundred metres above the current treeline when viable seeds and suitable microsites for germination were available. However, dense vegetation cover at lower elevations and winter mortality at higher elevations particularly limited early recruitment. Post-dispersal predation, species, and provenance also affected emergence and early establishment. This study demonstrates the importance of understanding multiple abiotic and biotic drivers of early seedling recruitment that should be incorporated into predictions of treeline dynamics under climate change.

## Introduction

Plant species are responding to recent global temperature increases^1^ by shifting their ranges as populations track their fundamental niche^2,3^. There is increasing evidence for climate-induced latitudinal range shifts via increased shrub abundance in circumarctic tundra ecosystems^4–6^ and elevational shifts of shrubs and trees in mountainous regions^7–11^. Treeline position, i.e. the range limit of forest ecosystems, is widely considered temperature sensitive and is thus expected to respond to climate warming^12–15^. However, global treeline dynamics are often modulated by regional-scale drivers such as historical land use changes^16^ and biotic interactions^17^. Hence, treeline responses to global warming vary among locations and are often asynchronous with the rate of climate change^17–21^.

Climate change-induced range expansion of treeline populations also depends on successful recruitment, which requires dispersal of viable seeds followed by successful establishment of individual propagules^22^. In treeline ecotones, viable seed availability commonly declines with elevation^13,23^ due to lower abundance of seed bearing trees and less frequent mast years, i.e. synchronous production of large seed crops^24–26^. Biotic interactions, such as pre-dispersal predation, may further constrain seed productivity at treeline^27^, impacting future treeline range expansion. Successful recruitment also depends on the availability of suitable microsites that provide the necessary conditions for emergence and establishment of seedlings^28,29^. Seed bed quality is determined by a complex interplay of abiotic and biotic factors such as microclimatic conditions, the presence of neighbouring vegetation, and herbivory^30^. Abiotic factors are considered key drivers of seedling recruitment in climatically harsh environments^23^. Early establishment is particularly limited by temperature and water availability^31–33^, but other abiotic factors, such as snow cover duration and desiccating winds, may also affect seedling recruitment^34–36^.

Biotic interactions can be equally or even more important than abiotic factors in determining seed bed conditions^37^. Microsite cover effects can be highly complex, with neighbouring vegetation positively or negatively affecting tree seedlings depending on vegetation type, species, demographic state, and prevailing weather conditions^29,38^. On the one hand, neighbouring vegetation can facilitate recruitment by sheltering seedlings from adverse climate effects, seed predators, and herbivores^23,28,39,40^. On the other hand, a dense vegetation cover can impede seedling emergence and establishment by competing for light, water and nutrients, exerting allelopathic effects, and preventing seeds from reaching a suitable seed bed^41–45^. The stress-gradient hypothesis predicts that these biotic interactions vary with abiotic conditions^46^. Therefore, it is expected that competition dominates at lower elevations with relatively low levels of environmental stress, whereas facilitative interactions prevail in more stressful environments at higher elevations^40^. Furthermore, seed predation and herbivory are other important biotic constraints on seedling recruitment at tree species’ upper range limits^47–50^. Dense vegetation may additionally promote post-dispersal predation by creating preferred microhabitats and foraging areas for seed predators and herbivores^51,52^. Thus, a large number of abiotic and biotic factors shape microsite conditions indicating the need for a better understanding of the interplay among these different drivers of seedling recruitment.

Seedling recruitment depends not only on environmental but also on genetic factors acting on seed availability, germination, growth, and survival. Individual tree species are adapted to different elevation ranges, reflecting different temperature sensitivities^13^. In particular, tree species are adapted to different ranges of germination temperatures^31^. Recruitment at treeline is thus likely to vary among species. Furthermore, provenance tests have revealed high genetic diversity and site-specific adaptations in conifer species^53–55^. Physiological and growth adaptations of high-elevation provenances to cold climate conditions are known to exist for later-stage seedlings and adult trees^56^. Provenance may also be important in early life stages of seedlings but has rarely been evaluated (but see^57^).

Major bottlenecks to tree seedling recruitment clearly occur in early life stages, yet analysing abiotic and biotic key players in this process remains a scientific challenge. While the influence of individual abiotic or biotic factors on seedling recruitment in treeline ecotones has been tested experimentally in several studies^31,32,42,58,59^, interactions among several abiotic and biotic factors have rarely been addressed^37,50,60^. In the European Alps, there exists, to the best of our knowledge, no experimental study testing the relative importance of multiple abiotic and biotic factors on early tree seedling recruitment along an elevation gradient across the treeline ecotone. Here, we investigated seedling emergence and early establishment of two conifer species, *Larix decidua* MILL. (European larch) and *Picea abies* (L.) KARST. (Norway spruce), below, at, and above treeline in the Swiss Alps. In two consecutive years, we sowed seeds of low- and high-elevation provenances of the two species into plots with intact vegetation and into plots where surface vegetation was removed to create open microsites to test the influence of seed availability, microsite cover, and provenance on early seedling recruitment. Moreover, herbivore exclosures allowed us to test the influence of seed predators and herbivores. We tested hypotheses addressing biotic and abiotic drivers of early seedling recruitment, specifically, predicting that: (i) emergence would be limited by both seed availability and harsh environmental conditions at high elevation, and thus would decline with increasing elevation and distance from the current treeline; (ii) recruitment would be greater at open microsites than in closed, intact vegetation at lower elevations, but the opposite effect would occur at high elevations where neighbouring vegetation shelters seedlings from adverse environmental conditions; (iii) there would be a negative effect of seed predation and herbivory on seedling recruitment, but this effect would be less pronounced at open microsites; and (iv) at and above treeline, recruitment would be greater for *L*. *decidua* than for *P*. *abies* and for high-elevation provenances of both species.

## Results

### Seedling emergence

**Figure 1:**
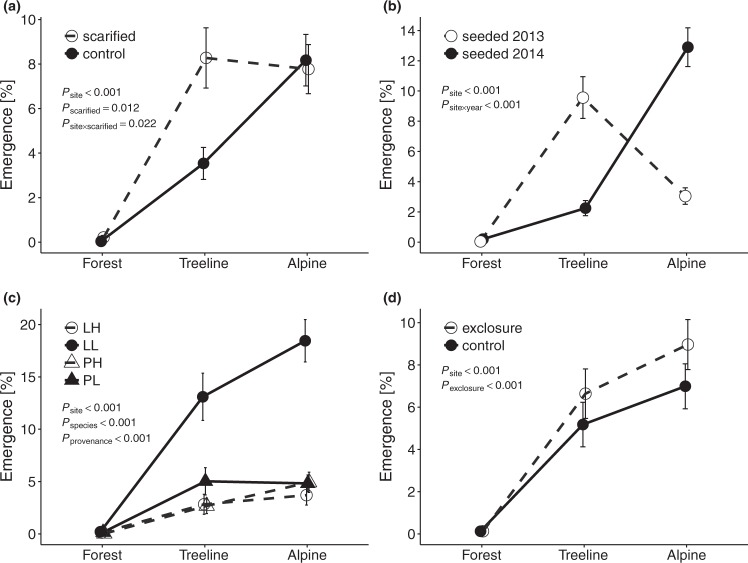
Effects of scarification treatment (**a**), seeding year (**b**), provenance and species (**c**), and herbivore exclosure treatment (**d**) on seedling emergence at the forest, treeline, and alpine sites. LL: low-elevation provenance of *L*. *decidua*; LH: high-elevation provenance of *L*. *decidua*; PL: low-elevation provenance of *P*. *abies*; PH: high-elevation provenance of *P*. *abies*. *P*-values indicate significant effects and interactions from likelihood ratio tests of mixed-effects models. Error bars indicate ± 1 standard error of trait means.

Naturally emerged seedlings were found only at the lowest study site (referred to as forest site) and only during the baseline census in June 2013, when 15 *L*. *decidua* seedlings and 7 *P*. *abies* seedlings were observed. Emergence in seeded plots was greater than in unseeded control plots (*n* = 960; *t* = −11.36; *P* < 0.001). Germination of experimentally sown seeds was highest at the uppermost site (alpine site), where 981 seedlings emerged (8.0 ± 0.8%, values represent mean ± 1 standard error of the percentage of viable seeds per seeded subplot, for absolute numbers see Supplementary Table S1a), followed by the mid-elevation site (treeline site) with 734 seedlings (5.8 ± 0.8%), and lowest at the forest site, where only 12 seedlings emerged (0.1 ± 0.04%; *P*_site_ < 0.001; Table 1; Fig. 1). Seedling emergence was greater in scarified plots than in plots with intact vegetation (5.4 ± 0.6% vs. 3.9 ± 0.5%; *P*_scarified_ = 0.012; Fig. 1a), in particular because more seedlings emerged in scarified plots than in plots with intact vegetation at the treeline site (8.0 ± 1.4% vs. 3.5 ± 0.7%) but not at the alpine site (8.0 ± 1.2% vs. 8.0 ± 1.5%) and at the forest site (0.2 ± 0.1% vs. 0.0 ± 0.0%; *P*_site × scarified_ = 0.022; Fig. 1a). Moreover, the positive effect of scarification on emergence was more pronounced in 2013 (5.2 ± 1.0% vs. 3.1 ± 0.5%) than in 2014 (5.6 ± 0.9% vs. 4.6 ± 0.8%; *P*_scarified × year_ = 0.041). Total emergence was similar in both years of seeding, 4.2 ± 0.6% in 2013 and 5.1 ± 0.6% in 2014 (*P*_year_ = 0.703). In 2013, emergence was more than three times as high at the treeline than at the alpine site (9.5 ± 1.4% vs. 3.0 ± 0.5%), whereas in 2014, emergence at the alpine site was more than six times as high than at the treeline site (13.1 ± 1.4% vs. 2.0 ± 0.5%; *P*_site× year_ < 0.001; Fig. 1b). Emergence was about twice as high for *L*. *decidua* than for *P*. *abies* (6.3 ± 0.7% vs. 2.9 ± 0.4%; *P*_species_ < 0.001) and almost three times as high for low-elevation provenances than for high-elevation provenances (6.9 ± 0.7% vs. 2.4 ± 0.3%; *P*_provenance_ < 0.001). Emergence of *L*. *decidua* seedlings from low-elevation provenance (10.6 ± 1.3%) was three to five times as high than emergence of high-elevation provenance seedlings (2.1 ± 0.4%) and from both *P*. *abies* provenances (3.3 ± 0.6% vs. 2.5 ± 0.4% for low- vs. high-elevation provenances; *P*_species × provenance_ < 0.001; Fig. 1c). Protection against seed predators and herbivores resulted in an overall increase in emergence from 4.0 ± 0.5% to 5.2 ± 0.6% (*P*_exclosure_ < 0.001; Fig. 1d). This effect was slightly stronger in 2013 than in 2014 (*P*_year × exclosure_ = 0.037) and the increase was more pronounced for low- than for high-elevation provenance seedlings (*P*_provenance × exclosure_ = 0.005).

**Table 1:** Effects of experimental site, scarification, seeding year, species, provenance, and herbivore exclosure treatment (exclosure), as well as their interactions, on seedling emergence, first and second winter survival, and seedling height.

	Seedling emergence (*n* = 1,727)	1^st^ winter survival (*n* = 408)	2^nd^ winter survival (*n* = 236)	Seedling height (*n* = 54)
Site	75.898^***^	26.408^***^	10.689^**^	21.219^***^
Scarified	6.308^*^	18.638^***^	12.460^***^	2.678
Year	0.146	0.521	—	—
Species	24.687^***^	2.412	3.416	—
Provenance	38.326^***^	3.779	0.367	—
Exclosure	15.490^***^	0.253	—	—
Site × Scarified	7.623^*^	2.398	1.021	1.975
Site × Year	92.573^***^	29.399^***^	—	—
Site × Species	1.515	10.249^**^	0.002	—
Site × Provenance	0.724	0.001	0.002	—
Site × Exclosure	0.033	0.134	—	—
Scarified × Year	4.175^*^	0.019	—	—
Scarified × Species	0.730	0.264	—	—
Scarified × Provenance	0.471	6.909^**^	—	—
Scarified × Exclosure	0.061	1.262	—	—
Year × Species	1.584	2.333	—	—
Year × Provenance	1.021	4.199^*^	—	—
Year × Exclosure	4.371^*^	4.695^*^	—	—
Species × Provenance	16.473^***^	0.148	—	—
Species × Exclosure	1.069	1.051	—	—
Provenance × Exclosure	7.896^**^	0.404	—	—

Values and symbols are χ^2^-values and significances, respectively, from likelihood ratio tests of mixed-effects models. Significance levels: *P < 0.05; **P < 0.01; ***P < 0.001. Degrees of freedom: df = 1 for all factors except for site and its interactions in seedling emergence (df = 2). The forest site was excluded from survival and growth trait models because of very low seedling recruitment.

### Winter survival and growth

**Figure 2:**
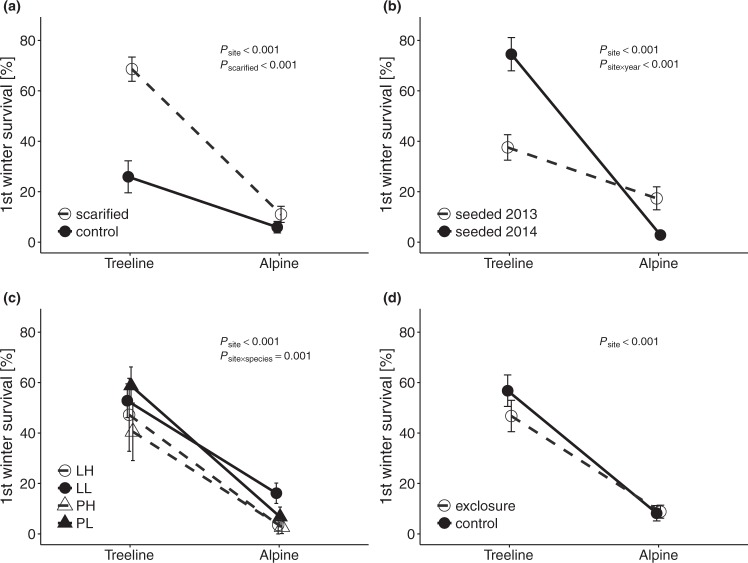
Effects of scarification treatment (**a**), seeding year (**b**), provenance and species (**c**), and herbivore exclosure treatment (**d**) on first winter survival of seedlings at the treeline and alpine sites. Abbreviations: see Fig. 1. *P*-values indicate significant effects and interactions from likelihood ratio tests of mixed-effects models. Error bars indicate ± 1 standard error of trait means.

The fraction of seedlings that survived the first winter was lower at the alpine site (8.5 ± 2.0%) than at the treeline site (51.5 ± 4.4%, *P*_site_ < 0.001; Table 1; Supplementary Table S1b; Fig. 2). First winter survival was generally higher in scarified plots than in plots with intact vegetation (*P*_scarified_ < 0.001; Fig. 2a). Furthermore, first winter survival at the treeline site was higher in winter 2014/2015 than in winter 2013/2014 (*P*_site × year_ < 0.001; Fig. 2b). More *L*. *decidua* than *P*. *abies* seedlings survived the first winter at the alpine site, whereas there was no difference in survival at treeline (*P*_site × species_ = 0.001; Fig. 2c). Slightly more low-elevation provenance seedlings of both species survived the first winter compared to high-elevation provenance seedlings (*P*_provenance_ = 0.052; Fig. 2c). The difference in first winter survival between scarified plots and plots with intact vegetation was greater for seedlings from high-elevation provenances than for seedlings from low-elevation provenances (*P*_scarified × provenance_ = 0.009). First winter survival was higher for seedlings from low- than from high-elevation provenances seeded in 2014, whereas there were no provenance differences for seedlings seeded in 2013 (*P*_year × provenance_ = 0.040). Slightly more seedlings from 2013 outside of herbivore exclosures than inside exclosures survived the first winter, whereas there was no difference for seedlings from 2014 (*P*_year × exclosure_ = 0.030). However, herbivore exclosure alone did not influence first winter survival (*P* > 0.1; Fig. 2d).

**Figure 3:**
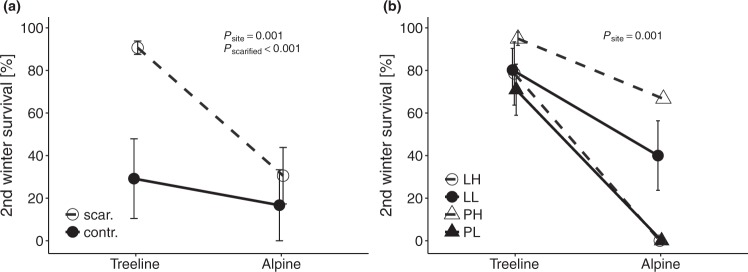
Effects of scarification treatment (**a**) and provenance and species (**b**) on second winter survival of seedlings at the treeline and alpine sites. Abbreviations: see Fig. 1. *P*-values indicate significant effects and interactions from likelihood ratio tests of mixed-effects models. Error bars indicate ± 1 standard error of trait means.

**Figure 4:**
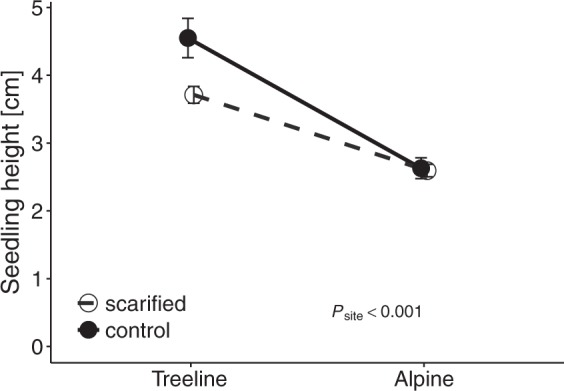
Effects of scarification treatment on seedling height at the treeline and alpine sites. *P*-values indicate significant effects and interactions from likelihood ratio tests of mixed-effects models. Error bars indicate ± 1 standard error of trait means.

Similar to first winter survival, fewer seedlings survived the second winter at the alpine site (25.9 ± 10.3%) than at the treeline site (79.2 ± 6.0%; *P*_site_ = 0.001; Table 1; Supplementary Table S1c; Fig. 3). Second winter survival was generally higher in scarified plots than in plots with intact vegetation (*P*_scarified_ < 0.001; Fig. 3a). The survival of *P*. *abies* seedlings during the second winter was slightly greater than that of *L*. *decidua* seedlings (*P*_species_ = 0.065), whereas provenance did not influence survival (*P*_provenance_ > 0.1; Fig. 3b). After the second growing season, seedlings were significantly taller at treeline (3.8 ± 0.1 cm) than at the alpine site (2.6 ± 0.1 cm; *P*_site_ < 0.001; Table 1; Fig. 4). However, there were no differences in seedling height with respect to scarification treatment (*P* > 0.1).

### Climate and soil temperatures

In 2013, mean summer air temperature (JJA, i.e. June, July, and August) at the Stillberg climate station was 1.2 °C above average, but in 2014 it was 0.8 °C below the 30-year average (1981–2010) of 9.5 °C (Supplementary Fig. S2). Summer 2015 was the second warmest summer on record, with a mean summer temperature 2.3 °C above average (Supplementary Fig. S2). In the summers of 2013 and 2015, precipitation was 79% and 75% of the average precipitation sum of 448 mm in the 1981 to 2010 period. Conversely, the precipitation sum in summer 2014 was 39% above average. Mean snow depth between 1 November and 30 April was similar for both winters (106% and 108%, respectively, of the 1981 to 2010 average of 78 cm; Supplementary Fig. S3).

Over the three years, summer soil temperatures (JJA) logged at each site were consistently lowest at the forest site and highest at the alpine site (Supplementary Table S2). Furthermore, mean summer soil temperatures in scarified plots were 0.4 °C higher than in plots with intact vegetation at the treeline site, whereas the corresponding temperature difference at the alpine site was 0.2 °C. Based on soil temperature measurements, the forest and treeline sites were snow free at the beginning and middle of May, respectively, in both years, whereas the alpine site was snow free at the beginning of June in 2014 and in mid-May in 2015 (Supplementary Table S3). The growing season started within two to three days after snowmelt and ended in mid-October in all three years (Supplementary Table S3).

## Discussion

In our study, tree seedlings emerged at and well above the current treeline but only when viable seeds were sown in suitable microsites for germination. Seedling emergence and early establishment were reduced in plots with intact vegetation below and at treeline but not at the alpine site. Species, provenance, and post dispersal predation further affected emergence. Winter survival and growth of seedlings were lower at the alpine than at the treeline site.

### Seed availability and microsite conditions determined recruitment

Observations of naturally emerged seedlings at the forest site in 2013 and the fact that this was a good seed crop year in the region for both species (personal communication A. Burkart, 2016) suggested that seeds were naturally available and able to emerge, as has been previously shown for other nearby subalpine forest stands^25^. The complete lack of natural recruitment at the treeline and alpine sites, and pronounced experimental seeding effects, however, indicated that emergence was limited by viable seed availability at and above treeline. Reduced viable seed availability in treeline ecotones may be a consequence of lower quality of high elevation seeds or increasing distance to seed bearing trees^24^.

After experimental seed addition, more *L*. *decidua* and *P*. *abies* seeds successfully germinated at the alpine site than at the treeline and forest sites (Table 1; Fig. 1), indicating that seedling emergence is possible several hundred metres above the current treeline and thus is not limited by the environmental conditions at high elevation if viable seeds are available. In contrast to air temperature that generally decreases with elevation, our records of summer soil temperatures showed the highest averages and largest fluctuations at the alpine site and in scarified plots at treeline (Supplementary Table S2 and Fig. S4). These higher soil temperatures in open microsites, due to enhanced surface heating by direct insolation^61^, may have promoted emergence at higher elevations.

The lack of emergence at the forest site (Table 1; Fig. 1a) was likely due to the tall and large-leafed herbaceous *Adenostylion* understorey vegetation, which may have directly competed for light, water, and nutrients, but probably also reduced seed bed temperatures. Although our experimental design did not allow us to disentangle the contributing factors, the proliferating growth of this understorey vegetation over the summer season likely impeded survival of the few naturally emerged seedlings and germination of sown seeds in both control and scarified plots. Indeed, a dense cover of understorey vegetation has been considered an important recruitment-limiting factor in other subalpine conifer forests^62,63^ as well as in boreal forests^37^. These understorey limitations are particularly pronounced where favourable microsites on rotten logs, stumps, and root-soil-plates are absent^64^.

Although a considerable number of sown seeds germinated at treeline, emergence and winter survival were reduced in plots with intact vegetation cover compared with in scarified plots (Table 1; Figs 1a, 2a, 3a). Whereas many other studies reported positive effects of neighbouring vegetation at treeline (e.g.^29,45,65,66^), our results indicate predominantly negative effects on early seedling recruitment at the treeline site. This site is characterised by a dense dwarf shrub layer, a vegetation type that has been shown to impair tree seedling recruitment by competing for water, nutrients, and light^43,67^. Although we did not measure resource levels, and thus cannot determine the underlying mechanisms, seedlings in control plots tended to grow taller (Fig. 4), suggesting increased competition for light in vegetation-covered plots^68^. In line with our findings, microhabitat comparisons in a Pyrenean alpine treeline ecotone revealed that microsites with dense dwarf shrub layers were not suitable for the recruitment of shade-intolerant *Pinus uncinata* seedlings^28^. Moreover, in alpine treeline ecotones in southern Norway and the French Alps, competition by herbaceous neighbouring vegetation has been suggested to reduce seedling emergence of *P*. *abies*^37^ and *L*. *decidua*^38^. These dense subalpine grassland vegetation types – as well as dwarf-shrub layers – in the Alps are very different from the low-stature alpine tundra vegetation at more continental treelines in the Rocky Mountains^38^, where positive biotic interactions prevail^29,69,70^. Thick and dense moss layers below the dwarf-shrub canopy at our treeline site may have further constrained seedling emergence by preventing seeds from reaching the soil surface and by soil moisture deficits during drier periods^71^. However, depending on moss thickness, moisture content, and moss species, moss seed beds can also facilitate seedling recruitment^72–74^. Furthermore, negative allelopathic effects of dwarf shrubs and mosses cannot be completely ruled out^44,75–77^.

While removal of vegetation cover enhanced early seedling recruitment at treeline, there was no difference in emergence between scarified and vegetation-covered plots at the alpine site (Table 1; Fig. 1a), indicating that there was no net effect of neighbouring vegetation on recruitment at this elevation. Indeed, the absence of tall dwarf shrubs, the scarce low-stature vegetation, and a relatively high proportion of bare mineral soil in this alpine meadow may have provided suitable seed beds for germination. Similarly, Munier, *et al*.^50^ related the reduced positive effects of substrate disturbance on tree seedling recruitment at alpine sites to the habitat-specific high proportion of disturbed ground and moss seed beds. Although in our treeline ecotone net negative biotic interactions seem to have decreased with elevation, we did not detect facilitative interactions at the alpine site. This finding, which is in contrast to those of other studies (e.g.^69^) and to predictions of the stress-gradient hypothesis^40^, may be due to the structure of the particular alpine plant community, the specific susceptibilities of the studied seedling species^38^, or the specific location of the study sites on a northeast-facing mountain slope, where heat stress and desiccation may be less important factors^78^.

Besides microsite, prevailing weather conditions can also influence seedling recruitment^29^. Indeed, the reduced emergence at the alpine site in the warm and dry summer 2013 compared to in 2014 (Table 1; Fig. 1b) may have been due to excessive soil warming and desiccation of seed beds. These effects have been shown to inhibit germination and cause damage to freshly emerged seedlings^23,31,65,79,80^. This suggests that extreme weather patterns, such as summer droughts, which are expected to become more frequent under future climate change, might strongly affect seedling recruitment. As our observations were based on a short experimental period, longer-term monitoring may improve our understanding of seed source and recruitment mechanisms and their impacts on treeline dynamics. In the long run, population modelling^81,82^ suggests that other effects, such as dispersal distance and differences in recruitment success, may be more important in determining future treeline position. Nevertheless, the pronounced emergence after experimental seed addition in a warm and dry year as well as in a cool and wet year suggests that a recruitment pulse is possible once viable seeds reach suitable microsites for emergence at higher elevation, opening the potential for treeline expansion.

### Seed source and post-dispersal predation modified recruitment success

*Larix decidua* had greater emergence success at and above treeline than *P*. *abies* (Table 1), which is in line with its higher upper range limit and greater tolerance for low temperatures^31,83^. In contrast to our hypothesis, more *L*. *decidua* seedlings from low- than from high-elevation provenance emerged at and above treeline (Table 1). This may be explained by its 16% greater seed mass compared to that of the high-elevation provenance (Table 2a), which might indicate a maternal effect or genetic differentiation among provenances^84^. The better performance of low-elevation provenances persisted over the first winter but diminished over the second year, with only 15% of the seedlings surviving for another full year (Supplementary Table S1). The role of provenance is thus likely to change over time. Similarly, a study on early seedling recruitment of *Picea engelmannii* showed that low-elevation provenances were selected for better initial survival and high-elevation provenances for tolerating harsher climate conditions in later stages of seedling establishment^57,60^. Likewise, a study with transplanted four-year-old *P*. *abies* seedlings indicated higher growth rates and frost tolerance levels for seedlings from high- compared to those from low-elevation provenances near treeline^85^. The similar responses of seedlings from high-elevation provenances and from the low-elevation *P*. *abies* provenance in our study suggested similar environmental sensitivities in early stages of seedling establishment. However, elevation-specific adaptations, such as different tolerance levels for frost or snow breakage^86^, may become more apparent in later stages of seedling establishment.

**Table 2:** Locations of the seed provenances (a) and experimental sites (b).

	Longitude [°N]	Latitude [°E]	Elevation [m a.s.l.]	Aspect	Slope [°]	Collection year	TGW [g]	Viability [%]	Elevation range of species [m a.s.l.]
(a) Provenance									
LL	46.699	9.709	1350	SW	—	1995	8.5	28	600–2100
LH	46.509	9.849	1760	SE	—	1970	7.3	11	600–2100
PL	46.917	9.785	1000	S	—	1985	6.8	74	500–1800
PH	46.734	9.849	1960	SW	—	1983	6.8	61	500–1800
(b) Experimental site									
Forest	46.777	9.868	1930	NE	25–30	—	—	—	—
Treeline	46.774	9.866	2090	NE	35–40	—	—	—	—
Alpine	46.769	9.862	2410	NE	25–30	—	—	—	—

TGW: seed mass in thousand grain weight. Viability: seed viability. LL: low-elevation provenance of *L*. *decidua*; LH: high-elevation provenance of *L*. *decidua*; PL: low-elevation provenance of *P*. *abies*; PH: high-elevation provenance of *P*. *abies*.

The overall positive effect of herbivore exclosures on emergence is in line with our hypothesis and other studies, which showed that post-dispersal predation and herbivory can constrain seedling recruitment in treeline ecotones^48,49^. Although we did not directly observe seed and seedling predation and cannot disentangle the two, empty seed coats in experimental plots as well as seedlings damaged by herbivory indicated that both forms of predation were present. Moreover, damage to seedlings inside herbivore exclosures suggests that invertebrate predation also played a role, as has been described for other forest and treeline ecosystems^50,74,87^. The observed preference of seed predators for the low-elevation *L*. *decidua* provenance (Table 1) may be explained by its greater seed weight (Table 2a), as predators have been shown to prefer heavier, nutrient-rich seeds^23,88^. Contrary to our expectation, predation had similar effects on open and vegetation-covered microsites, suggesting that either vegetation did not influence predation, or different groups of predators profited equally from both microsite types. Furthermore, the effects of predation and herbivory may be overestimated in seeding experiments, like the one presented here, because the high density of seeds or seedlings may attract herbivory^89,90^, whereas natural seedlings at low density would be less affected^69^. Thus, additional studies of natural seedlings are needed to quantify the importance of post-dispersal seed predation and herbivory effects for treeline dynamics.

### Seedling survival and growth declined with elevation

In contrast to emergence, winter survival was lower at the alpine than at the treeline site (Table 1; Figs 2, 3) and thus was possibly limited by adverse climate conditions at high elevations during snow free periods between the fall census and the spring census of the following year. Indeed, records of soil temperatures were lower at the alpine than at the treeline site in these periods (Supplementary Fig. S4). Similarly, other studies showed that winter survival of tree seedlings is primarily determined by climate conditions during snow-free periods in early and late winter^91^. While low temperature may have directly impacted survival, the combination with bright sunlight causing photoinhibition may have additionally increased seedling mortality at the high elevation site. Both effects can impose limitations on early tree seedling survival at alpine treelines^28,35,69,92^. The considerably lower first winter survival at treeline in winter 2013/2014 than in the following winter (Table 1; Fig. 2b) may have been a consequence of the slow formation of an insulating snow cover (Supplementary Fig. S3) leaving seedlings not well protected against freezing events in late fall. Similarly, Batllori, *et al*.^28^ observed high mortality of *P*. *uncinata* seedlings in a winter with a shallow snow cover. Besides its strong influence on seedling survival, snow cover duration may also have influenced seedling growth. The observed decline in seedling height with increasing elevation (Table 1; Fig. 4) is in line with the observation of reduced growth rates due to shorter growing seasons at higher elevation^13,86^. In addition, Zurbriggen, *et al*.^58^ suggested that declining tree seedling growth at higher elevation may be due to reduced soil nutrient availability.

First winter survival was considerably lower than second winter survival at the treeline site (Supplementary Table S1), whereas a similar comparison for the alpine site was impossible due to high first winter mortality. Seedlings that survived the first winter mostly also survived the following summer at both sites (Supplementary Fig. S5). These results confirm the common observation that the first winter is an important bottleneck for seedling recruitment, see Körner^13^ and references therein. Thus, high winter mortality in alpine environments can strongly affect overall recruitment and contributes to the complex puzzle of multiple abiotic and biotic factors determining regeneration in treeline ecotones.

## Conclusions

This study provides experimental evidence for the successful emergence and early establishment of tree seedlings at and above the current treeline when viable seeds reach suitable microsites for germination. While dense understorey and dwarf-shrub vegetation may prevent infilling of open subalpine forests below and at treeline, recruitment above treeline is spatially and temporally restricted to suitable microsites and climatically favourable years. Our findings demonstrate the importance of multiple abiotic and biotic drivers of early seedling recruitment in the treeline ecotone that should be considered when predicting treeline dynamics under climate change.

## Methods

### Study species and provenances

The two study species, *L*. *decidua* and *P*. *abies*, are among the most important treeline-forming conifers in the European Alps, with *L*. *decidua* mostly restricted to the European Alps and *P*. *abies* common in the subalpine and boreal zones of Eurasia^93^. In Switzerland, the elevation range of *L*. *decidua* is 600 to 2,100 m a.s.l., whereas *P*. *abies* has a slightly lower elevation range of 500 to 1,800 m a.s.l.^83^. For each species, we used seeds from a low- and a high-elevation provenance, located within 30 km of the study area (Table 2a). Seeds were procured from the Swiss Federal Institute for Forest, Snow and Landscape Research WSL (Birmensdorf, Switzerland) and stored at 5 °C prior to sowing. Seed viability was determined by direct germination tests under laboratory conditions.

### Experimental design

In summer 2013, three experimental sites were established along an elevational gradient across an alpine treeline ecotone located on a northeast-exposed slope in the Dischma valley, Davos, Switzerland (Table 2b; Supplementary Fig. S1). The lowest study site (forest site) is located at 1,930 m a.s.l., below the treeline and close to the regional upper range limit of *P*. *abies* and *L*. *decidua*, in a subalpine larch-spruce forest (*Larici-Picetum*) with tall and large-leafed herbaceous understorey vegetation (predominantly *Adenostylion*; canopy height approx. 50–100 cm). The mid-elevation site (treeline site) is located at 2,090 m a.s.l., at the current treeline, and is dominated by dense dwarf shrub vegetation (predominantly *Rhododendro-Vaccinietum*; canopy height approx. 50 cm) interspersed with low-tree islands of *Pinus cembra* and *Pinus mugo*. The soil was covered by a 10–15 cm thick moss layer at this site. The uppermost site (alpine site) is located at 2,410 m a.s.l., approximately 300 m above treeline, in an alpine meadow with a vegetation height of approx. 5–15 cm (*Caricetum curvulae*).

The three experimental sites were set up following the standard protocol of the global G-TREE initiative^94^. In a split-plot design, 20 whole plots (224 cm × 45 cm) were established at each site. They were completely randomly assigned to the 2 × 2 treatment combinations of the main factors seeding and scarification (i.e. seeding and scarification, seeding only, scarification only, and full control), resulting in five replications per main treatment combination. Distances between whole plots were at least 0.5 m, but usually between 1 m and 10 m. Each whole plot was divided into 16 split-plots (22.5 cm × 28 cm; referred to as subplots), to which treatment combinations of the four additional two-level factors species, provenance, herbivore exclosure, and seeding year were randomly assigned. Overall, this resulted in 960 split-plots (3 sites × 20 main plots per site × 16 split-plots per main plot). Plot setup and seeding treatment applications were staggered in time at the three sites (forest, then treeline, then alpine) to reflect the natural difference in growing season start at the three elevations. At the beginning of the experiment in summer 2013, natural recruitment and vegetation cover were assessed in each subplot. Thereafter, we applied the scarification treatment by removing vegetation, plant litter, mosses, and lichens from the plot surfaces with a hand-cultivator, but leaving roots and non-organic material in the soil. With this treatment we simulated soil disturbance and created open microsites, which is an established method to study biotic interactions with neighbouring vegetation^37,95^. While vegetation cover regrew within several weeks at the forest site, we did not observe significant regrowth of vegetation cover over the experiment duration in scarified plots at treeline and at the alpine site although the plots were scarified only once. Immediately after scarification, 200 seeds were spread evenly on each of the respective subplots. A second seeding treatment was applied to a different set of subplots at the beginning of the following growing season in spring 2014. In both years, immediately after seeding, herbivore exclosures made of durable and stable metal cages (45 cm × 28 cm, mesh size 2 × 4 cm) were installed on half of the subplots for the duration of the growing season. Each cage covered two adjacent subplots. We expected that the cages would exclude small mammals and birds, whereas burrowing animals, such as voles, and invertebrates were not prevented from entering the plots (personal communication M. Schütz, 2013). Fences were installed at each site to protect experimental plots from grazing and trampling by cattle and horses.

Seedling recruitment was assessed by counting the seedlings in each subplot at the beginning and end of the growing seasons in 2013, 2014, and 2015. All seedlings were individually marked to avoid double counts. Emergence was defined as the percentage of germinated seeds at the end of the first growing season (i.e. three to four months after sowing). First and second winter survival were defined as the percentage of surviving seedlings between the fall census and the spring census of the following year. Seedling height was measured with a hand ruler as the total length from the original emerging point to the apical meristem of approximately 15-month-old seedlings at the end of the growing seasons 2014 and 2015. In subplots with more than ten seedlings, only ten haphazardly chosen seedlings were measured. Seedling growth was defined as the average seedling height per subplot after two growing seasons. Maximum seedling height per subplot was also tested and showed similar results because of small height variation among seedlings (data not shown).

Daily values of mean air temperature, precipitation, and snow depth were measured at the Stillberg climate station at 2,090 m a.s.l. located at the Stillberg Long-Term Ecosystem Research site^96^ approx. 50 m from the treeline site. At each site, two to five temperature loggers (iButton; Maxim Integrated Products, Sunnyvale, CA, USA) recorded soil temperatures at a depth of 5 cm in vegetation-covered plots, as well as in plots where surface vegetation was removed. The beginning and end of the growing season were defined as the dates when soil temperature rose for the first time above (beginning) or fell below (end) 3.2 °C for two contiguous days^14,97^. Additionally, the dates of the first snow cover in autumn and snowmelt in spring were derived from soil temperature measurements and defined as the dates when daily temperature fluctuations stopped and soil temperature remained at 0 °C (snow cover), and when daily temperature fluctuations restarted (snowmelt).

### Statistical Analyses

A two-sided *t*-test was applied to compare emergence in seeded versus non-seeded plots. Linear and generalised linear mixed-effect models (LMMs and GLMMs)^98^ were used to analyse the response of seedling recruitment (emergence, winter survival, and growth) to the investigated abiotic and biotic factors and their interactions. Due to the almost complete lack of natural germination, non-seeded plots were omitted from these analyses. The forest site was excluded from survival and growth trait models because of very low seedling recruitment. GLMMs for seedling emergence and first winter survival contained site, scarification treatment, seeding year, species, provenance, and herbivore exclosure treatment as fixed effects. Because of the small data set for second winter survival, it was not possible to test the influence of seeding year and herbivore exclosure treatment, as well as their interactions. All three GLMMs used binomial distributions. Seedling height was modelled using a LMM with the fixed effects site and scarification treatment and their interaction. All models accounted for spatial correlation among plots and subplots by including plot and two subplot-structures as random effects. Significance of model factors was determined by likelihood ratio tests, and fixed effects that did not significantly improve the model fit were eliminated. All models were fitted using standard procedures for model diagnostics^99^ with the lme4-library (version 1.1–13)^100^ in R 3.3.3^101^.

### Data availability

Climate data are archived in the EnviDat Digital Repository: 10.16904/envidat.43^96^ and 10.16904/envidat.42^102^. All other data generated during and/or analysed during the current study are available from the corresponding author upon reasonable request.

## Electronic supplementary material

Supplementary Information
