# Supplementary material for: Biotic and abiotic drivers of tree seedling recruitment across an alpine treeline ecotone
Source: Sci Rep. 2018 Jul 18;8:10894. doi: 10.1038/s41598-018-28808-w (PMC6052039; doi:10.1038/s41598-018-28808-w)
Supplement: Supplementary file 1 — Supplementary Information [file 41598_2018_28808_MOESM1_ESM.pdf]

Biotic and abiotic drivers of tree seedling recruitment across an alpine treeline ecotone

Esther R. Frei [1,2*], Eva Bianchi[1,3], Giulietta Bernareggi[1,4], Peter Bebi[1], Melissa A. Dawes[1,2], Carissa D. Brown[5], Andrew J. Trant[6], Steven D. Mamet[7], and Christian Rixen[1]

[1]WSL Institute for Snow and Avalanche Research SLF, Flüelastrasse 11, 7260 Davos Dorf, Switzerland

[2]Swiss Federal Institute for Forest, Snow and Landscape Research WSL, Zürcherstrasse 111, 8903 Birmensdorf, Switzerland

[3]Institute of Terrestrial Ecosystems, Department of Environmental Systems Science, ETH Zurich. Universitätstrasse 22, 8092 Zurich, Switzerland

[4]Dipartimento di Bioscienze, Università di Parma, Parco Area delle Scienze 11/A, 43124 Parma, Italy

[5]Department of Geography, Memorial University, 230 Elizabeth Avenue, St John's, NL, A1B 3X9 Canada

[6]School of Environment, Resources and Sustainability, University of Waterloo, 200 University Avenue West, Waterloo, ON, N2L 3G1 Canada

[7]Department of Soil Science, University of Saskatchewan, 51 Campus Drive, Saskatoon, SK S7N 5A8 Canada

*Correspondence: Esther R. Frei, e-mail: estherfrei@gmx.net

Supplementary Information

Table S1. Number of seedlings for emergence (a), first winter survival (b), and second winter survival (c), grouped by species and provenance per species for experimental site, scarification treatment, seeding year, and herbivore exclosure treatment.

	Total	*L. decidua*	*P. abies*	LL	LH	PL	PH
(a) Emergence							
Forest site	12	5	7	4	1	6	1
Treeline site	734	311	423	288	23	295	128
Alpine site	981	452	529	421	31	288	241
Scarified	997	438	559	398	40	366	193
Intact	730	330	400	315	15	223	177
Seeded 2013	804	345	459	324	21	317	142
Seeded 2014	923	423	500	389	34	272	228
Exclosures	983	434	549	404	30	355	194
Unprotected	744	334	410	309	25	234	176
(b) 1st winter survival							
Forest site	0	0	0	0	0	0	0
Treeline site	348	164	184	148	16	147	37
Alpine site	60	51	9	50	1	5	4
Scarified	344	173	171	157	16	131	40
Intact	64	42	22	41	1	21	1
Seeded 2013	302	132	170	121	11	135	35
Seeded 2014	106	83	23	77	6	17	6
Exclosures	236	119	117	112	7	97	20
Unprotected	172	96	76	86	10	55	21
(c) 2nd winter survival							
Forest site	0	0	0	0	0	0	0
Treeline site	227	104	123	96	8	94	29
Alpine site	9	7	2	7	0	0	2
Scarified	229	104	125	96	8	94	31
Intact	7	7	0	7	0	0	0
Seeded 2013	236	111	125	103	8	94	31
Seeded 2014	–	–	–	–	–	–	–
Exclosures	127	53	74	52	1	58	16
Unprotected	109	58	51	51	7	36	15

LL: *L. decidua* from low-elevation provenance, LH: *L. decidua* from high-elevation provenance; PL: *P. abies* from low-elevation provenance, PH: *P. abies* from high-elevation provenance.

Table S2. Monthly mean soil temperatures (mean ± 1 standard deviation) for the experimental sites from July 2013 to August 2015.

Year	Month	Soil temperature forest site [°C]	Soil temperature treeline site [°C]	Soil temperature alpine site [°C]
2013	July	10.3±1.1	–	–
	August	9.7±1.4	9.3±0.9	11.3±2.3
	September	7.0±1.6	6.8±1.2	6.6±2.5
	October	4.8±1.9	4.8±1.3	3.2±2.2
	November	1.5±0.9	2.1±0.5	0.7±0.1
	December	0.3±0.1	0.8±0.2	0.4±0.1
2014	January	0.4±0.0	0.8±0.0	0.3±0.0
	February	0.4±0.0	0.8±0.0	0.3±0.0
	March	0.4±0.0	0.8±0.0	0.3±0.0
	April	0.5±0.3	0.7±0.0	0.3±0.0
	May	4.7±2.1	3.3±2.0	0.7±0.7
	June	8.4±1.5	8.6±1.4	9.2±3.7
	July	8.7±1.1	9.4±1.1	9.9±2.5
	August	8.5±1.2	9.0±1.3	9.4±1.8
	September	7.3±1.2	7.2±1.3	6.9±1.7
	October	5.2±2.4	4.9±1.6	3.8±2.1
	November	1.9±0.7	1.9±0.3	0.6±0.1
	December	0.3±0.6	0.6±0.4	0.4±0.0
2015	January	0.2±0.1	0.3±0.1	0.3±0.0
	February	0.2±0.0	0.4±0.0	0.2±0.0
	March	0.2±0.0	0.4±0.0	0.2±0.0
	April	0.3±0.0	0.4±0.0	0.3±0.0
	May	4.6±2.2	3.0±2.2	2.7±3.1
	June	9.1±1.5	9.5±1.3	11.1±3.0
	July	11.5±1.2	12.4±1.1	16.3±2.3
	August	10.8±1.4	11.2±1.4	13.3±2.7

Table S3. Dates of springtime snowmelt, and beginning and end of growing season based on soil temperatures at the three experimental sites in 2013 – 2015. Dates are indicated as DOY (day of the year).

	2013			2014			2015		
Site	Snow-melt	Growing season start	Growing season end	Snow-melt	Growing season start	Growing season end	Snow-melt	Growing season start	Growing season end
Forest	NA	NA	285	123	126	296	124	126	NA
Treeline	NA	NA	298	133	136	297	132	134	NA
Alpine	NA	NA	284	153	155	295	131	133	NA

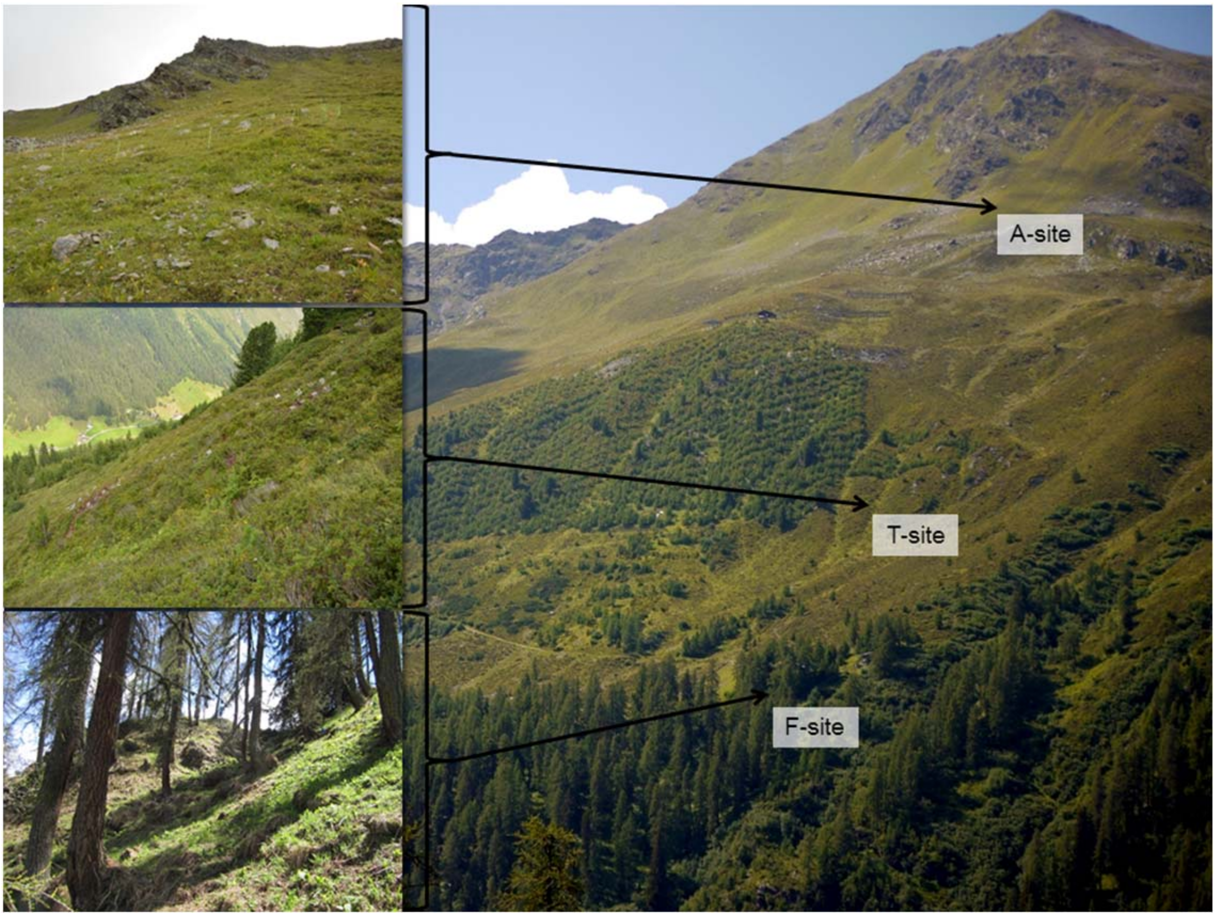

Figure S1. Location of the forest (1,930 m a.s.l.), treeline (2,090 m a.s.l.), and alpine (2,410 m a.s.l.) sites across the alpine treeline ecotone in the Dischma valley, Davos, Switzerland (Photos: M.A. Dawes, E.R. Frei).

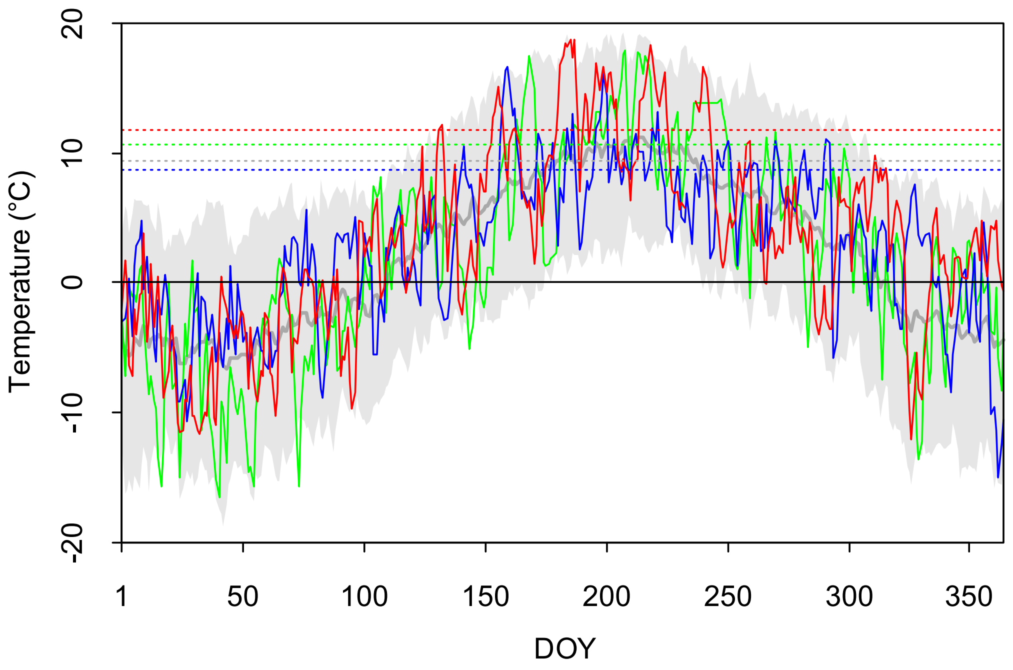

Figure S2. Air temperatures in the three study years 2013 (green), 2014 (blue), and 2015 (red) at the Stillberg climate station at 2,090 m a.s.l. (46.774 °N; 9.867 °E). Mean summer air temperatures (JJA) for each year are indicted by a dotted line in the respective colour. Average air temperatures for the 1975 – 2015 period are shown by the grey line with the grey shading indicating ± 2 standard deviations and the grey dotted line indicating the average (1975 – 2015) mean summer air temperatures (JJA).

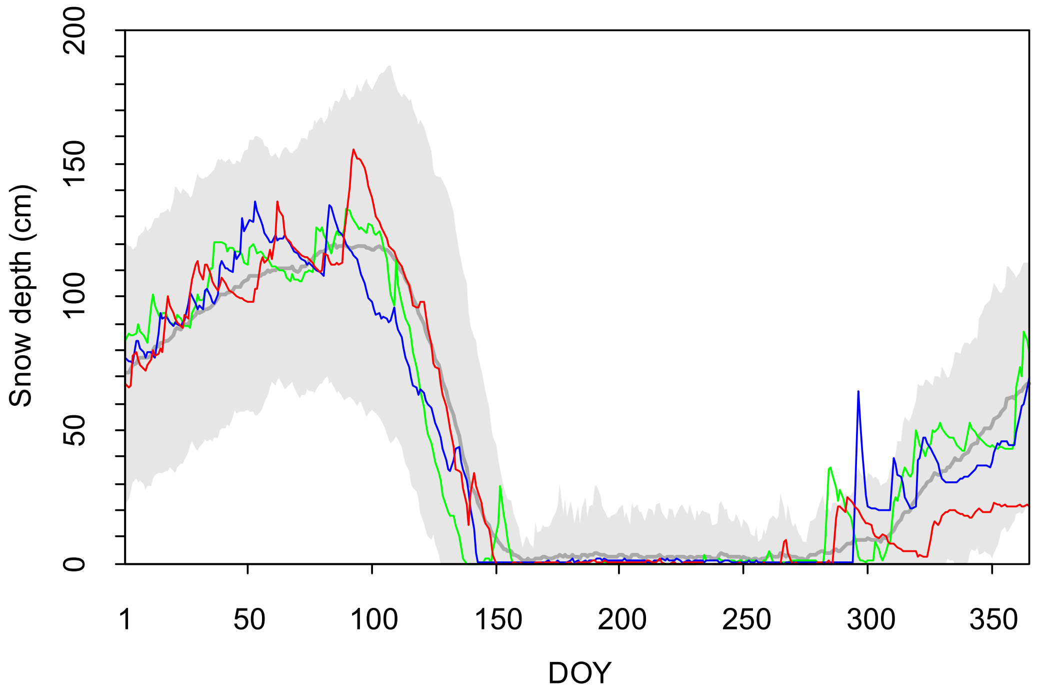

Figure S3. Snow depth in the three study years 2013 (green), 2014 (blue), and 2015 (red) at the Stillberg climate station at 2,090 m a.s.l. (46.774 °N; 9.867 °E). Average snow depth for the 1975 – 2015 period is shown by the grey line with the grey shading indicating ± 2 standard deviations.

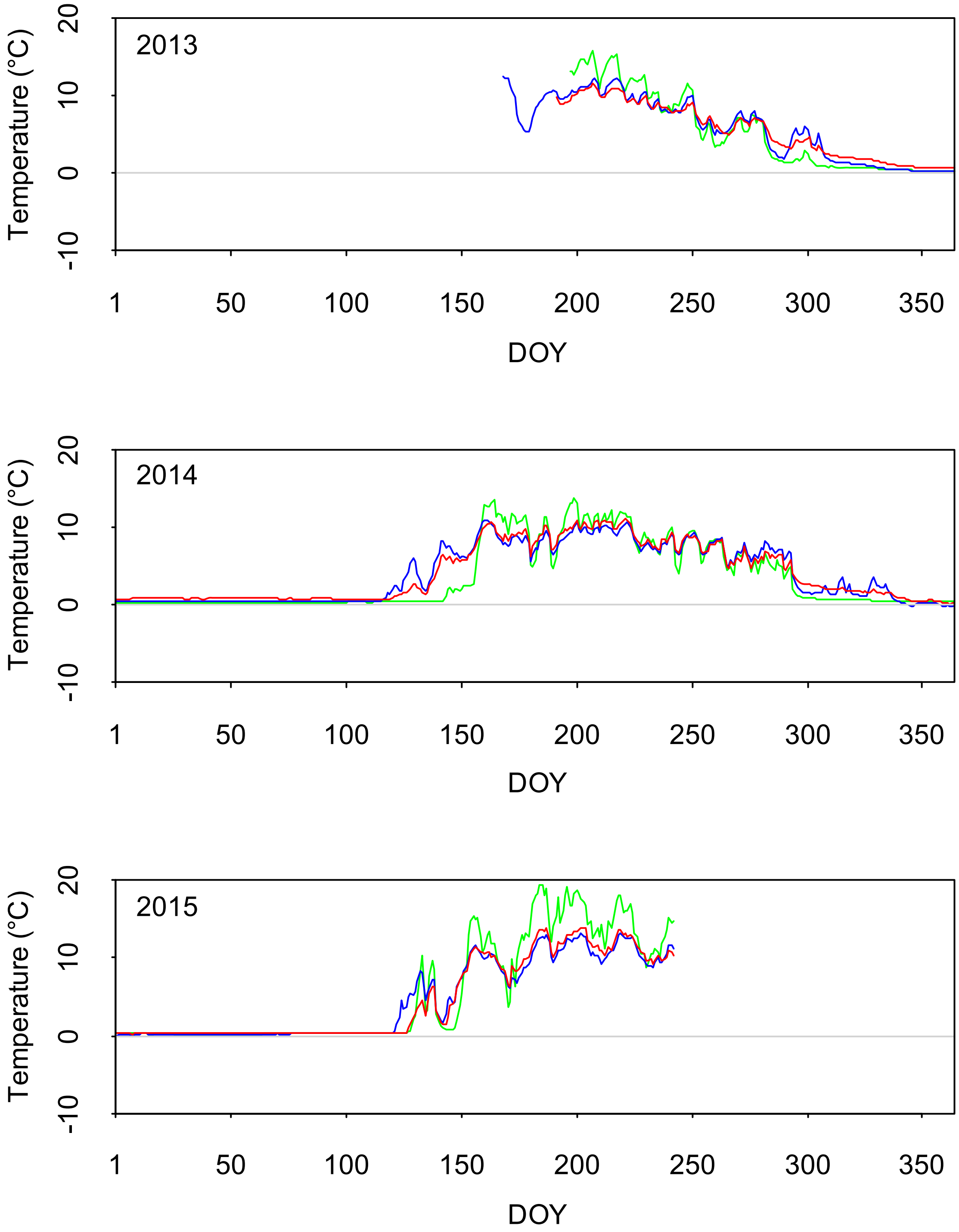

Figure S4. Daily mean soil temperatures for the alpine (green), treeline (blue), and forest (red) sites from July 2013 to August 2015 measured by iButtons (Maxim Integrated Products, Sunnyvale, CA, USA) at 5 cm soil depth.

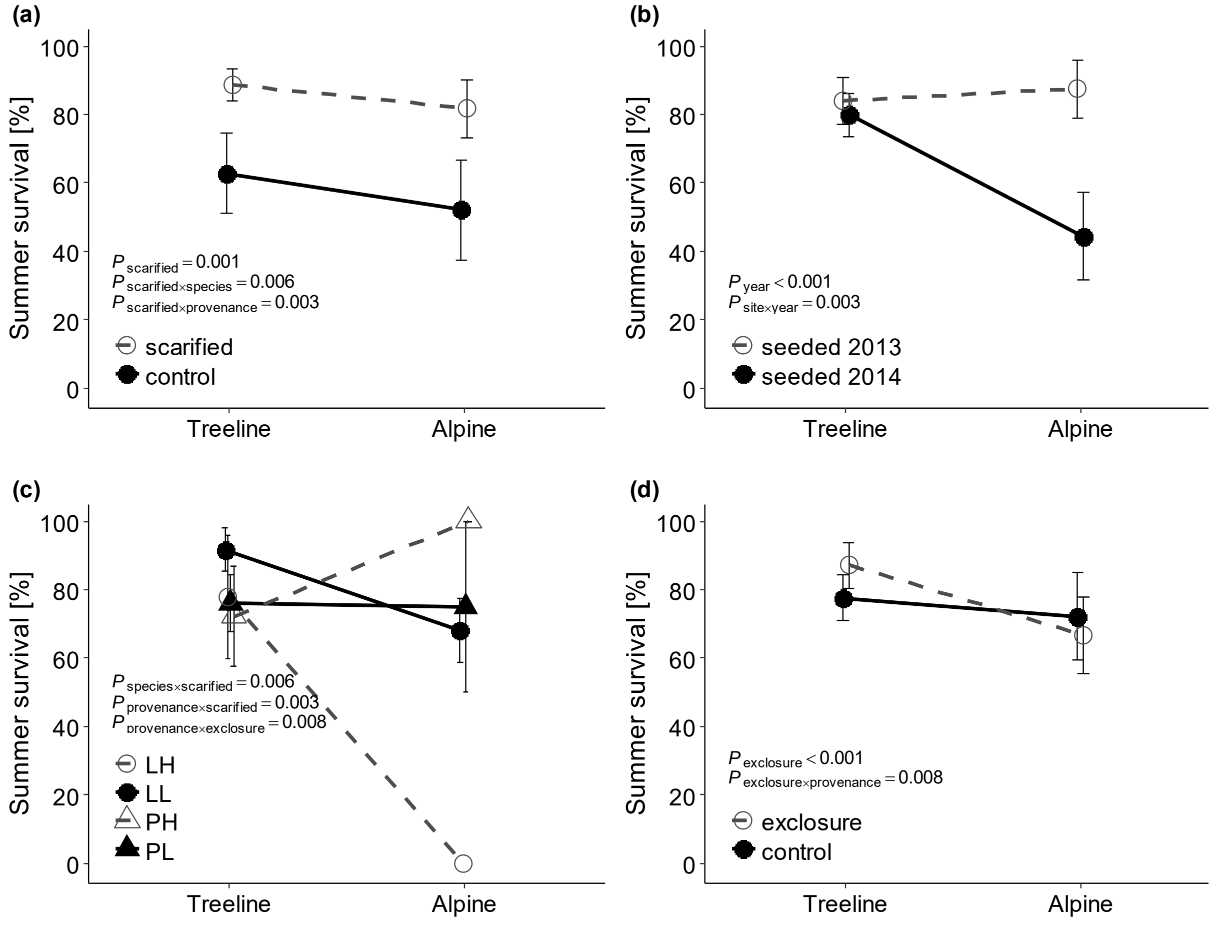

Figure S5. Effects of scarification treatment (a), seeding year (b), provenance and species (c), and herbivore exclosure treatment (d) on summer survival of seedlings at the treeline and alpine sites (n = 355). LL: low-elevation provenance of *L. decidua*; LH: high-elevation provenance of *L. decidua*; PL: low-elevation provenance of *P. abies*; PH: high-elevation provenance of *P. abies*. *P*-values indicate significant effects and interactions from likelihood ratio tests of mixed-effects models (data not shown). Error bars indicate ± 1 standard error of trait means.
